# Impact of trayless dining intervention on food choices of university students

**DOI:** 10.1186/s13690-018-0301-5

**Published:** 2018-09-24

**Authors:** Janani Rajbhandari-Thapa, Katherine Ingerson, Kristina H. Lewis

**Affiliations:** 1 Department of Health Policy and Management, College of Public Health, University of Georgia, 211 B Wright Hall (HSC), Athens, GA 30602 USA; 2 Food Services Department, University of Georgia, Athens, GA 30602 USA; 3 Department of Epidemiology & Prevention, Division of Public Health Sciences, Wake Forest Baptist Medical Center, Winston-Salem, NC USA

**Keywords:** Obesity, Behavioral economics, Food behavior, Low cost, Trayless dining

## Abstract

**Background:**

Students live outside of their family homes for the first time in college and are expected to make their own decisions regarding dietary choices. College food environment could be a major determinant of dietary intake and is of importance in relation to obesity. This research determines the impact of removing cafeteria trays on student’s food choice.

**Method:**

A quasi experimental pre-post research with control treatment was conducted in university dining halls. The participants were the dining hall patrons at a large public university in Southern US, spring 2015. The dining hall trays were removed from the intervened dining hall for five consecutive days during regular university session. Outcome measures of food choice were collected by observing tray waste before and after the tray removal in the intervened dining hall with parallel observation in the control dining hall. Difference-in-difference analysis was done to find the intervention effect.

**Results:**

A total of 3153 trays were observed (*N* = 1564 in control and *N* = 1589 in intervention dining). Removal of trays resulted in a significant decrease in the total number of lunch plates (1.76 vs 1.66 servings, *p* < .006), drink glasses (1.32 vs 1.02 servings, *p* < .0001), dishes with leftovers (0.56 vs 0.39 serving, *P* < .001), and lunch plates with leftovers (0.51 vs 0.35 servings, *p* < .005).

**Conclusions:**

Student food choices can be affected by removing trays from dining halls, specifically favoring fewer beverages, and without sacrificing salad consumption. Studies with more precise measures of tray waste are needed to understand the direct effect on energy and nutrient consumption.

## Background

For many students, college represents the first time they have lived outside of their family homes, and are expected to make their own decisions regarding many daily activities, including dietary choices. Late adolescence, the time when most students enter college as freshmen, is marked by a sense of independence and autonomy, as well as a propensity toward stress, depression, and risk-taking behaviors, as one transitions from being viewed as a child to an adult [1]. Combining these psychological factors with a food environment that makes abundant food available through university dining halls may predispose college students to experience weight gain. In fact, the familiar concept of the “freshman 15” has been borne out in studies, though the scientific evidence for freshman students gaining 15 pounds in the first year of college is divided is mixed [2].

Food environment is a major determinant of dietary intake and is of importance in relation to obesity. Obesity in the US is an ongoing public health concern. As of 2012, 34.9% of adults were obese and 68.5% were overweight or obese [3]. The costs of obesity in the US have been estimated at $147 billion in medical costs plus $42.8 billion in productivity losses annually (2008 US$) [4]. Obesity, with some exceptions, can largely be attributed to positive energy balance [5]: a situation in which energy consumed is greater than energy expended, leading to weight gain. On average, diet quality in the US is far from ideal and contributes to a net positive energy balance: people consume more energy than they need to sustain their activity level [6]. This might be specifically true for college students who have increasing demand for passive time and an increased access and independence in food choice decision.

The university setting may offer a unique opportunity to provide a food environment that promotes healthful dietary habits that lead to decreased weight gain in adulthood. As students learn to regulate their own food intake and make dietary choices in environments with numerous options, colleges and universities can facilitate healthful choices in many ways, including creating “built environments” in their cafeterias that allow for better portion control. Environmental or policy level changes that encourage young people to make better choices when dining outside of their homes is an identified approach to prevent obesity [7]. In this, previous research has shown that information provision through signage and nutrition labels and increased availability of healthy foods improves dietary intake in university settings [8]. Previous research has also shown found that the quantity of food consumed by the residents of a nursing home varied according to their perception of the quantity served. However, impact of trayless dining on food behavior has not been studied at the level of the current study. In this study, we tested if removing trays from a university dining hall can improve dietary habits. Removing trays changes the choice architecture of university dining halls. Choice architecture is the design of different ways in which choices can be presented to consumers. The change of that presentation can impact consumer decision-making. By removing the trays from a dining hall, the consumer is impacted in the amount of choices he can carry at one time. The impact of that change within the dining hall setting is considered in this study. Several choice architecture changes in the university dining hall setting have been tried and evaluated with mixed findings [9]. Many dining halls have been moving towards trayless dining for sustainability reasons, but the effects in terms of impact on food choices behavior have not been evaluated. This study, conducted in conjunction with the University’s Office of Sustainability, relied on a two-group pre-post design to evaluate the effect of trayless dining on student food choices.

## Methods

### Setting

This study was conducted in spring 2015 within two dining halls at a public university in the Southern US with a total undergraduate student population of approximately 26,000 individuals. The two dining halls in this study serve 3600 students each in an average day during the semester.

### Study design and intervention

The current research captured a unique opportunity to study impact of the university’s sustainability initiative on students’ food choices. The University’s Office of Sustainability was evaluating one week of trayless dining in one of the five university dining halls to test effectiveness of trayless dining on reducing food, water, and energy waste. We worked with the University’s Office of Sustainability and the University Dining Services to study the impact of its trayless dining intervention on students’ food choice behavior. Trayless dining is a change in choice architecture and could have impact on food choices beyond the impact on water and energy use. This study was possible through the researchers’ collaboration with the university food services’ registered dietitian. The dietitian provided information about the trayless dining intervention and intervention timeline to allow for the design of the study.

The study used selection of dishes as a proxy for selection of food items. Four types of dishes namely lunch plates, salad bowls, dessert plates, and drinking glasses were used as proxy for the types of foods selected. While there is the possibility that dishes are not being used for what they are meant to be used for, we believe there are two reasons why this might not be common in this research setting, 1) the layout of the dining hall is such that each food service area, entrée, salad, and dessert are separate from one other, so it would be inconvenient for a student to take salad bowl and go to the entrée section to get entrée or vice versa, and 2) the use of used dishes including drink glasses for refills is strictly discouraged for hygiene purposes. This limits the possibility of 1) dishes being inter-used between items, for example dessert and entrée, and 2) one drink glass being used for several servings of drink. Further, if a student returned for seconds or a refill, requiring new plates each time allows number of refills to be reflected in the total number of plates observed in this study.

The study design was a two group pre-, post- design with data collection from one control and one treatment dining hall during a baseline and an intervention period. The “intervention” or trayless dining hall was selected by the administration because it had the most inconspicuously-located tray stack among the University dining halls. The students were not notified by the University in advance about the trays being removed. The control cafeteria was selected based on the control dining hall’s similarity with the intervention dining hall with respect to size, operation hours, food being offered, layout, management team, and student body being served. The tray and dish size in the control dining hall was also the same as in the intervention dining hall. The two dining halls were located on the same side of the campus and as such served the same student group. The design of the study also captures seasonal effects on the outcome.

Baseline data was collected in February 2015. Either a registered dietitian or a research assistant (dietary intern) trained by the registered dietitian was present in the dish return area of the control and intervention dining halls for one-hour (11 am to 12 pm) during the lunchtime meal, for five consecutive weekdays prior to tray removal. The research assistants stood by the return dish rack to observe the trays as they were placed on the return rack. After one week, the trays were removed from the intervention cafeteria, and the process of data collection in both cafeterias was repeated, again during the lunchtime meal from 11 am to 12 pm, for five consecutive weekdays.

Data was collected using the established tray waste record sheet [10] method modified for this study. The tray waste record sheet was a printed form with boxes to record the total number of lunch servings, drink servings, salad servings, and dessert servings measured using respective dishes as proxies for number of servings of lunch entrée, drink, salad, and dessert respectively. The available drink options in the dining halls included fruit juices, milk, and water. The form also had options to record the amount of leftover (quarter leftover, half left over, or not eaten at all) of the entrée, salad, and dessert. The leftovers were observed in reference to a standard serving size for entrée, salad, and dessert as advised by the dining hall’s registered dietitian. This serving size was also the standard used to estimate food preparation volume and count servings of each food item served. The interns were trained about the full serving size for each of the dishes by the registered dietitian. Furthermore, the dining hall discourages patrons to take food outside of the dining hall, which limits the chances of consumption being un- or under- observed. Data were collected by the research assistants using direct observation of the trays. The data collection form was developed from the Quarter Waste Method, which is a validated method used to observe plate waste in a school lunchroom setting that could be used to generate an accurate measure of tray waste. It is a cost-effective, reliable and accurate visual method [10].

### Outcome variables

The primary outcome variables for this study are: 1) number of lunch entrée servings, 2) number of drink servings, 3) number of salad servings, 4) number of dessert servings, 5) number of lunch entrées, salads, and dessert servings with at least a quarter leftover, and 6) number of lunch entrées servings with at least a quarter left over. The first four outcome variables were chosen to give us an understanding of how tray removal altered food choices and drink consumption as measured using dishes as a proxy for food type selected. The first four measures rely on the assumption that the dish was used for specific foods they were provided for. For example: dessert plates were used for desserts. The latter two outcome variables were chosen to give us an understanding of how tray removal might impact food waste.

### Data analysis

The data was analyzed using SAS 9.3. Summary statistics across both dining halls for the pre- and post-intervention periods were calculated. Multivariate regression was used to estimate the impact of intervention on the six outcome variables. Each of the six outcome variables were regressed on a post-intervention variable of interest, which is a binary variable (1 if post intervention and 0 otherwise), and dummies for days of the week. Days of the week fixed effects were used to capture differences in day that could theoretically affect food behavior patterns, such as different students attending the dining hall, differences in the menu, differences in student mood (“It’s Monday…” vs “TGIF”), etc. The model was run separately for the treatment and control dining hall.

To test if the change in measurement variables differed between the treatment and control dining halls, the data was pooled and a multivariate regression was run with addition of an interaction term between the post-intervention period and the treatment dining hall (1 if post-intervention in treatment dining hall, 0 otherwise). The coefficient of the interaction term provided a difference in difference estimates of relative change between the control and intervention dining hall. This allows determination of whether the changes in the intervention dining hall are statistically different from those in the control dining hall.

## Results

### Summary statistics

A total of 3153 trays were observed over a period of two weeks (one week of pre-intervention and one week of post-intervention) in two dining halls (*N* = 1564 in control and *N* = 1589 in intervention). Summary statistics of the observed outcome measures is shown in Table 1. As shown in Table 1, the control and intervention dining halls were very similar at baseline with respect to the number and types of servings (lunch, drink, salad, dessert) being returned at the end of the lunchtime meal. The control and intervention dining halls were also similar with respect to leftovers.

**Table 1:** Summary statistic of the number (No.) of outcome measures

	Outcome measures	Control		Intervention		*P* value
		Mean	S.D	Mean	S.D	
Pre	No. of lunch servings	1.83	(1.02)	1.76	(0.97)	0.161
	No. of drink servings	1.39	(0.86)	1.32	(0.95)	0.124
	No. of salad servings	0.16	(0.43)	0.12	(0.38)	0.029
	No. of dessert servings	0.03	(0.18)	0.06	(0.27)	0.004
	No. of dish with at least a quarter leftover	0.62	(0.71)	0.56	(0.68)	0.088
	No. of entrée with at least a quarter leftover	0.55	(0.64)	0.51	(0.63)	0.312
Post	No. of lunch servings	1.92	(1.00)	1.66	(0.88)	0.000
	No. of drink servings	1.50	(0.87)	1.02	(0.80)	0.000
	No. of salad servings	0.12	(0.37)	0.14	(0.43)	0.269
	No. of dessert servings	0.03	(0.19)	0.04	(0.20)	0.528
	No. of dish with at least a quarter leftover	0.60	(0.69)	0.39	(0.55)	0.000
	No. of entrée with at least a quarter leftover	0.54	(0.64)	0.35	(0.53)	0.000

Number of servings is based on number of dishes used for each type of food items, assuming one dish is equal to one serving

### Regression outcomes

Multivariate regression based estimates on the outcome measures for the control and intervention dining halls are shown in Table 2. In the control dining hall there was a significant pre-to-post increase in drink servings and a significant decrease in salad servings. Meanwhile, in the intervention dining hall there was a significant decrease in five outcome measures after tray removal: 1) number of lunch servings, 2) number of drink servings, and 3) number of dessert servings, 4) number of servings (lunch entrée, salad, dessert) with at least a quarter leftover, and 5) number of entrée with at least a quarter leftover. There was an insignificant increase in number of salad servings in the intervention dining hall. The number of servings are based on number of dishes used for each type of food items.

**Table 2:** Estimates for change in outcome measures due to tray removal

	Control dining hall		Intervention dining hall		Diff-in-diff estimate	
	β	*p* value	β	*p* value	β	*p* value
No. of lunch servings	0.088	0.086	**−0.100**	0.031	**−0.188**	0.006
No. of drink servings	**0.108**	0.014	**−0.299**	<.0001	**−0.407**	<.0001
No. of salad servings	**−0.048**	0.017	0.021	0.309	**0.070**	0.015
No. of dessert servings	0.003	0.757	**−0.024**	0.039	−0.027	0.076
No. of dishes with leftover	−0.021	0.561	**−0.176**	<.0001	**−0.153**	0.001
No. of entrée with leftover	−0.008	0.811	**−0.162**	<.0001	**−0.152**	0.005

The first four columns of the tables are the estimated results for the control (first two column) and intervention (second two column) dining hall regressed separately. The estimates shown are for the binary post intervention variable. The difference in difference estimate on the relative change between the control and intervention dining hall is shown in the last two columns

The difference in difference estimates (in Table 2) show a significant decrease in the following outcome variables in the intervention cafeteria, relative to control: total number of lunch servings, total number of drink servings, servings (lunch entrée, salad, dessert) with at least a quarter leftover, and entrée with at least a quarter leftover. Tray removal also resulted in a significant increase in total number of salad bowls.

## Discussions

In this quasi-experimental study of trayless dining, we found that students in dining hall with no trays available at lunchtime self-served fewer servings (in fractions of a serving) of lunch entrée item and drink relative to students in a dining hall where trays remained in place. In addition, the students in dining hall with no cafeteria trays self-served more salad. These results, based on using different types of dishes as a proxy for types of food selected, suggest an increase in salad consumption and a decrease in lunch entrée and drink consumption in the trayless setting. The drink options in the dining halls included fruit juices, milk, and water using same type of glass; hence, the decrease in drink could have been either of the drink options available.

Results also found that cafeterias with no trays ended up with fewer leftovers meaning higher proportion of the lunch entrée, salad, and dessert servings were consumed. This clearly suggest that there was less food waste in cafeteria with no trays. The food waste observation was based on the standard serving size for each entrée as recommended by the dietician. There might have been some deviations in what the students actually serve in a self-service setting relative to the standard serving size. However, by design the study was close to a repeated measures design. There is high likelihood that same group of students were observed during the baseline and intervention period. As such, the deviations in serving size would have been systematic throughout the study.

The fewer number of lunch and dessert servings selected and higher number of salad servings selected combined with the decrease in servings of food wasted suggest in the trayless condition the students decreased the number of self-served food servings but increased the proportion of the self-served food that was consumed resulting in lower waste.

Environmental and policy changes that encourage people to make better food choices when dining outside of their home may be one approach to promote more healthful diets and prevent obesity [7]. Unfortunately, drastic or costly policies do not tend to gain traction in the political or private realm. Simple and subtle interventions (“nudges”) such as menu labelling with calorie information are more likely to translate directly into policy and be accepted by the American public [11]. A simple and low cost modification of the dining environment would be to remove trays from dining halls serving large number of patrons such as University dining hall. While a true list is not available, several U.S. colleges and universities have “gone trayless” as a cost saving, environmentally friendly measure to reduce food waste and water usage over the past few years. It is unknown, however, if this simple economical change might serve to trim the growing waistline of college students as they enter college commonly termed as the “freshman 15”.

These results clearly suggest that students take fewer servings during lunch time if trays are removed. However, studying the impact of a tray-removal policy on caloric consumption was beyond this study’s scope. One previous study reported a decrease in the percentage of diners who took salad by 65.2% but no decrease in the percentage of diners who took dessert [12]. The decrease in drink glasses that we observed, on the other hand, has more promise to indicate that such a policy could have a meaningful impact on caloric consumption. Sugar-sweetened beverage consumption is a key determinant of obesity in the United States [13], and may have a number of other negative impacts on health [14–16]. Although we cannot be certain whether drink glasses contained exclusively beverages high in sugar (as opposed to water), it is likely that calories from beverages decreased as a result of this intervention. We did not observe an effect of tray removal on number of dessert servings, unfortunately. A previous study also reported no decrease in the percentage of diners who took dessert [12]. However, the study was based on a smaller sample size (*N* = 417) compared to the current study and was conducted over a very short period of time, in two evenings one with tray service and one without. There are limited studies that look at the impact of the switch to trayless system on students’ food choice and dietary behavior of college students. In this study we measured the number of lunch servings, drink servings, salad servings and dessert servings separately in addition to observing plate waste by recording the amount leftovers to better understand the impact of trayless dining on food choice behavior.

Because sustainability and reducing food waste are important goals of the University, and served as the impetus for this natural experiment, it is important to review these impacts of tray removal as well. The Office of Sustainability estimates, based on this pilot, that approximately 18,849 gal of water and 107,142 pounds of food would be saved per semester due to elimination of waste from going trayless. Similar decreases in solid waste have been reported in switching from tray to a trayless system [17, 18].

Evidence-based, low cost, and easily scalable choice architecture changes guided by behavioral economics [19] have shown success in promoting selection and consumption of fruits and vegetables within existing school lunchroom infrastructure [20–23]. The application and effectiveness of such interventions are being tested in grocery stores and supermarkets [24]. In this, the results from this study are critical to consider the effect of changes in food environment on food choice in cafeteria settings. Switching from tray to trayless system could also be sustainable in settings similar to university dining halls such as senior centers, private cafeterias, and hospital cafeterias. A variety of populations are served in these settings, including low income beneficiaries of national food and health safety net systems. It is thus important to consider and evaluate the effectiveness of low cost choice architecture change such as going trayless in dining halls that serve large number of patrons.

### Limitations

The major limitation of our study is that we did not measure food / caloric intake directly, or look at consumption at the level of added sugars, saturated fats, or sodium. Using the different types of dishes used for entrée, salad, and dessert was not the best proxy for servings but given the short time to design the intervention with no funding available, this proxy measure provides insight on what is being consumed vs. trashed (beyond bulk consumption data at the preparation level). Nonetheless our measure is limited in that we did not have a thorough enough assessment to gather nutrient-level information. Another limitation of this study is that we did not examine whether students who took fewer servings of entrées, salads, desserts and drinks at lunchtime made substitutions or trade-offs in their consumption later, outside the university dining hall, with extra snacks or larger meals elsewhere. We feel that the chances of this happening in the present study are low, because of the nature of the university meal plan, which acts as a disincentive for students to eat anywhere outside of the dining halls, where all of their food is already paid for. However, the dining hall allows for multiple entries throughout the day. Therefore, patrons could come in for a snack at midafternoon.

## Conclusions

In the present-day U.S., many universities have gone trayless for sustainability reasons. Our results suggest that removing trays may reduce beverage consumption, and possibly overall caloric consumption, without decreasing intake of healthy food choices like salad, while reducing food and water waste [12]. More detailed studies that aim to estimate caloric consumption, as well as measuring certain key macronutrients such as fats and added sugars, are needed. In the meantime, for colleges and universities looking to make easy changes to choice architecture at minimal cost, several low cost interventions have been developed that may promote healthy consumption by students [25]. Such strategies may be useful in cafeterias settings that serve adult populations, including low income populations.
